# Pulmonary paracoccidioidomycosis showing reversed halo sign with nodular/coarse contour

**DOI:** 10.1590/0100-3984.2015.0071

**Published:** 2016

**Authors:** Rodolfo Mendes Queiroz, Michela Prestes Gomes, Marcus Vinicius Nascimento Valentin

**Affiliations:** 1 Documenta - Hospital São Francisco, Ribeirão Preto, SP, Brazil.; 2 Faculdade de Medicina de Ribeirão Preto da Universidade de São Paulo (FMRP-USP), Ribeirão Preto, SP, Brazil.

*Dear Editor,*

A 63-year-old man, living and working in urban area since his childhood, and smoking for 30 years. In 2012 he underwent investigation for chronic cough. At that same time, he reported gingival lesion. For a long time, he had the habit of weekly visiting rural areas for leisure and amateur fishing. The patient denied history of fever, weight loss or comorbidities. Blood counts since 2009 without any abnormalities.

**Figure 1:**
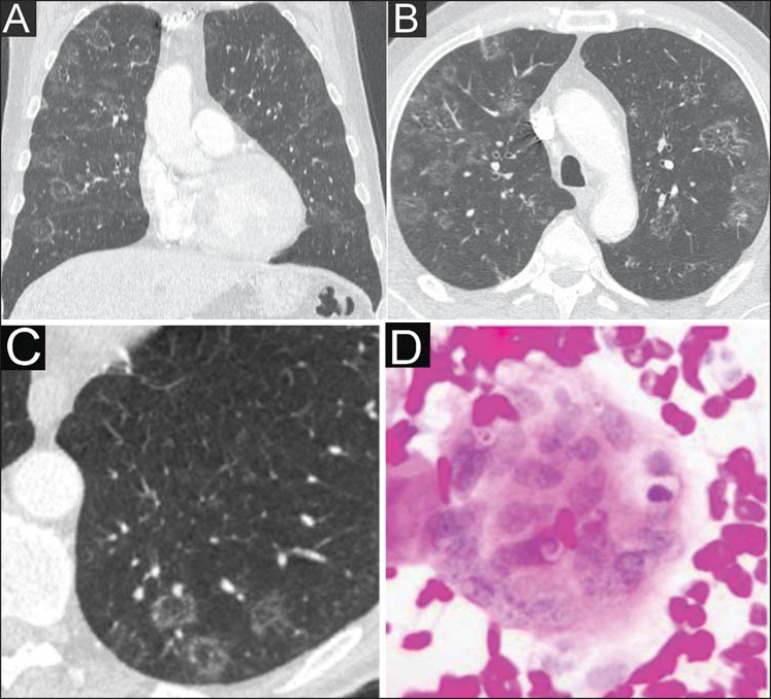
**A,B,C:** Computed tomography sections showing, principally, focal pulmonary ground glass opacities predominantly located in the middle fields, some of them either completely or partially surrounded by a thin and coarse consolidation ring representing the “reversed halo sign”. One can also observe some areas with subtle interlobular septa thickening. **D:** Histological slide of the biopsied gingival lesion demonstrating eosinophilic epithelial cells of the squamous and spinous layers; giant, “foreign-body” type cells containing isolated and clustered spherical fungi with double and birefringent membranes in association with inflammatory cells. The cytological diagnosis confirmed the presence of *Paracoccidioides brasiliensis*.

Chest computed tomography (CT) in December 30, 2013 showed focal pulmonary ground glass opacities predominantly in the middle fields, some of them completely or partially surrounded by a thin and coarse consolidation ring representing the "reversed halo sign". Other findings include some areas with subtle interlobular septa thickening (Figures 1A, 1B and 1C).

The gingival lesion, characterized by granular, erythematous ulceration with fine blood-red dots, compatible with a "mulberrylike" appearance, was biopsied.

Biopsy result: eosinophilic epithelial cells of the squamous and spinous layers, giant, "foreign-body" type cells containing isolated and clustered spherical fungi with double and birefringent membranes, in association with inflammatory cells. The cytological diagnosis confirmed the presence of *Paracoccidioides brasiliensis* (Figure 1D).

On February 2, 2015, post-itraconazol therapy chest CT demonstrated rare areas of hypoattenuation associated with fibrocicatricial septal thickening.

Paracoccidioidomycosis is the most common endemic systemic mycosis in the Latin America, caused by infection by inhalation of the dimorphic fungus *Paracoccidioides brasiliensis*^(1-7)^, a pathogen that is found only in Colombia, Argentina, Venezuela and principally subtropical regions in Brazil^(1,3,4,6)^. The incidence is high in men, rural workers^(1-7)^ aged between 30 and 60 years^(1,2,6).^

There are two presentations, as follows:

*Acute* - It is a rare presentation, affecting both male and female children and young adults, manifesting especially with hepatosplenomegaly, lymph nodes enlargement, weight loss and fever. Presentations on mucosas and skin are rarely found^(1-4,6)^.

*Chronic* - It represents 90-93% of cases, most of times in men aged above 30, affecting the lungs (90% of cases), with an insidious course after quiescent lesion reactivation in the lungs, progressing with pulmonary fibrotic lesions in 60% of the patients, with possibility of subsequent late respiratory failure. The course of the condition may be concomitant with involvement of the skin and mucosas (50-54% of cases), bones, adrenal glands, lymphatic system, digestive system and central nervous system; however, such sites are isolatedly affected in less than 10% of cases. Symptoms include chest pain; dyspnea, chronic cough either with or without expectoration; hemoptysis; weight loss and fever^(1-4,6)^.

Diagnostic confirmation is made by means of histopathological individualization of the fungus, mainly in bronchoalveolar lavage material and biopsies^(1,2)^.

Usually, in the chronic pulmonary presentation, CT findings tend to be symmetrical and bilateral, predominantly located in the lung bases, and include simple or complex patterns such as ground glass opacity (for example, "reversed halo sign"), consolidations, micronodules, nodules, masses, cavities, interlobular septa and peribronchovascular interstitium thickening, fibrotic lesions^(1-6)^.

"Reversed halo sign" consists in a focal ground glass opacity either completely or partially involved by a rounded area of consolidation, and is found in 10% of cases of paracoccidioidomycosis^(1,4,5,7)^. Despite its nonspecificity, some recent studies have associated the "reversed halo sign" and a nodular/coarse ring with infectious and non-infectious granulomatous diseases^(7,8)^.
